# IL4I1 Is Expressed by Head–Neck Cancer-Derived Mesenchymal Stromal Cells and Contributes to Suppress T Cell Proliferation

**DOI:** 10.3390/jcm10102111

**Published:** 2021-05-13

**Authors:** Alessio Mazzoni, Manuela Capone, Matteo Ramazzotti, Anna Vanni, Luca Giovanni Locatello, Oreste Gallo, Raffaele De Palma, Lorenzo Cosmi, Francesco Liotta, Francesco Annunziato, Laura Maggi

**Affiliations:** 1 Department of Experimental and Clinical Medicine, University of Florence, Largo Brambilla 3, 50134 Florence, Italy; alessio.mazzoni@unifi.it (A.M.); manuela.capone@unifi.it (M.C.); anna.vanni@unifi.it (A.V.); locatello.lucagiovanni@gmail.com (L.G.L.); oreste.gallo@unifi.it (O.G.); lorenzo.cosmi@unifi.it (L.C.); francesco.annunziato@unifi.it (F.A.); laura.maggi@unifi.it (L.M.); 2 Department of Biomedical, Experimental and Clinical Sciences, University of Florence, Largo Brambilla 3, 50134 Florence, Italy; matteo.ramazzotti@unifi.it; 3 Department of Otorhinolaryngology, Careggi University Hospital, Largo Brambilla 3, 50134 Florence, Italy; 4 Department of Internal Medicine, University of Genoa, 16126 Genoa, Italy; raffaele.depalma@unige.it; 5 Department of Immunology and Cell Therapies, Careggi University Hospital, Largo Brambilla 3, 50134 Florence, Italy; 6 Flow Cytometry and Immunotherapy Diagnostic Center and Immunotherapy, Careggi University Hospital, Largo Brambilla 3, 50134 Florence, Italy

**Keywords:** HNSCC, IL4I1, IDO1

## Abstract

Amino acids have a primary role in cancer metabolism. Beyond their primary biosynthetic role, they represent also an alternative fuel while their catabolites can influence the epigenetic control of gene expression and suppress anti-tumor immune responses. The accumulation of amino-acid derivatives in the tumor microenvironment depends not only on the activity of tumor cells, but also on stromal cells. In this study, we show that mesenchymal stromal cells derived from head–neck cancer express the amino acid oxidase IL4I1 that has been detected in different types of tumor cells. The catabolic products of IL4I1, H_2_O_2_, and kynurenines are known to suppress T cell response. We found that neutralization of IL4I1 activity can restore T cell proliferation. Thus, therapeutical strategies targeting enzymes involved in amino-acid catabolism may be helpful to contemporary block tumor cell migration and restore an efficacious anti-tumor immunity.

## 1. Introduction

IL-4 induced gene 1 (IL4I1) is an L-amino acid oxidase with immunomodulatory properties originally described in B cells and then in dendritic cells (DCs) and tumor-associated macrophages (TAMs). IL4I1 can suppress T cell proliferation via the enzymatic oxidation of phenylalanine, with the production of phenylpyruvic acid (PP) and H_2_O_2_. H_2_O_2_ is then responsible for the transient downregulation of TCR expression s [1]). IL4I1 is expressed also by some subsets of T cells, including Th17 and Treg [1,2]. In Th17 cells, IL4I1 acts as a mechanism to limit their proliferation via regulating the expression of the anti-proliferative molecule Tob1 [3]. Recent data have shown that different types of human cancer directly produce IL4I1, which generates metabolites that activate the Aryl hydrocarbon receptor (AhR). Indeed, in addition to phenylalanine, IL4I1 can also catabolize tyrosine and tryptophan, leading to the generation of hydroxyphenylpyruvc acid (HPP) and indole-3-pyruvic acid (I3P). I3P is responsible for AhR activation, that favors cancer cell motility and immune response suppression [4]. In addition to the direct inhibition exerted by tumor cells, the immune response is strongly counteracted also by cells of the tumor stroma. Mesenchymal stromal cells (MSC) are spindle-shaped cells commonly present at tumor sites, involved in the suppression of anti-tumor immune responses, thus favoring tumor escape [5]. Indeed, it has been shown that MSC can polarize macrophages to an anti-inflammatory phenotype, suppress the migration and maturation of dendritic cells (DC), and reduce NK cytotoxic capability. MSC also suppress T cell proliferation and cytokine production, promote the generation of Treg cells, and impair immunoglobulin synthesis by B cells [5]. One of the best-described molecular mechanisms responsible for MSC immune suppression involves the indoleamine dyoxigenase 1 (IDO1) enzyme. IDO1 catalyzes the rate-limiting reaction in the pathway that converts tryptophan in the final products kynurenines, which are involved in T cell proliferation arrest and apoptosis [6].

We have previously shown that MSC obtained from head–neck squamous cell carcinoma (HNSCC) inhibit T cell functions [7]. Moreover, we have shown that the transcriptional signature of HNSCC-MSC is strongly influenced by two cytokines commonly produced by anti-tumor T cells: IFN-γ and TNF-α [8]. In this study, we hypothesized that additional mechanism than IDO1 can be involved in the immunosuppressive activity of HNSCC-MSC. Starting from our previously described HNSCC-MSC transcriptome data, we demonstrated here that after in vitro stimulation with IFN-γ and TNF-α, HNSCC-MSC express the amino acid oxidase IL4I1. This enzyme has immunoregulatory properties and contributes to the HNSCC-MSC mediated T cell suppression. 

## 2. Materials and Methods

### 2.1. Patients

The procedures followed in the study were approved by the AOUC (Azienda Ospedaliero-Universitaria Careggi) Ethical Committee. HNSCC-MSC cell lines were derived from tumoral specimens from seven patients who underwent surgery and exhibited a typical surface markers phenotype as previously described (Mazzoni 2020). HNSCC-MSC lines expanded for less than 12 passages were used for experiments. Seven healthy donors were recruited to collect peripheral blood (PB). PBMNC were obtained from healthy donors following density gradient centrifugation using Lymphoprep. CD4+ T cells were then recovered by negative magnetic selection (Miltenyi Biotech, Bergisch Gladbach, Germany). The medium used for T-cell culture was RPMI 1640 (Seromed, Berlin, Germany), supplemented with 2 mM L-glutamine, 1% nonessential amino acids, 1% sodium pyruvate, 2 × 10^−5^ M 2-mercaptoethanol, and 10% FBS HyClone (Gibco Laboratories, Grand Island, NY, USA). HNSCC-MSC lines were maintained in DMEM high-glucose medium (EuroClone, Pero, Italy) supplemented with 15% FBS HyClone and 4 mM L-glutamine. For cytokine stimulation, HNSCC-MSC were treated overnight with IFN-γ (2 ng/mL, R&D systems, Minneapolis, MN, USA) and/or TNF-α (10 ng/mL, R&D systems, Minneapolis, MN, USA).

### 2.2. Microarray

Microarray data have been obtained from re-analysis of previously published data (Mazzoni 2020) and are available at [9].

### 2.3. Real-Time PCR

Total RNA was extracted by using the RNeasy Micro Kit (Qiagen, Hilden, Germany) and treated with DNase I to eliminate possible genomic DNA contamination. RNA reverse transcription was performed with Taqman Gold kit (Thermo Fisher Scientific, Massachusetts, USA). For gene expression analysis, total mRNA recovered from 2500 cells was reverse transcribed and then subjected to RT-PCR amplification. Ct values were then transformed into arbitrary units (AU) using a standard curve generated with a reference sample. Primers and probes used were purchased from Thermo Fisher.

### 2.4. Proliferation Assay

Scalar doses of HNSCC-MSC were stimulated overnight with IFN-γ (2 ng/mL) and TNF-α (10 ng/mL) in 96 well plates in DMEM medium. The day after the medium was replaced with fresh RPMI 1640 containing CD4+ T cells derived from PB of healthy donors (0.1 × 10^6^ cells/well) and agonist anti-CD3 and anti-CD28 antibodies (5 μg/mL each, BD Biosciences, NJ, USA), in presence or absence of catalase (1000 U/mL, Sigma Aldrich, Munich, Germany) or 1-methyl-tryptophan (500µM, Sigma Aldrich). On day three, cells were pulsed for 8 hr with 0.5 μCi of 3H-TdR (Perkin Elmer, Milan, Italy) and then harvested. Radionuclide uptake was measured by scintillation counting.

## 3. Results and Discussion

**Figure 1:**
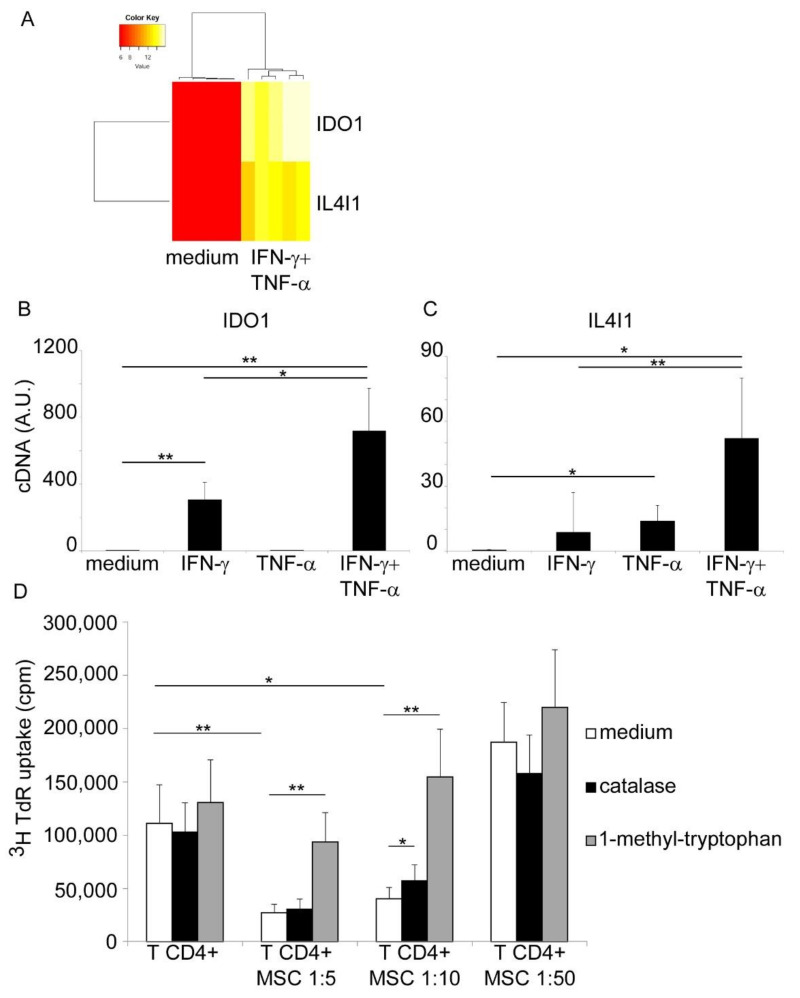
IL4I1 is expressed by HNSCC-derived MSC and controls T cell proliferation. (**A**) Hierarchical clustering-assisted heatmap analysis on selected genes from transcriptome data of five primary HNSCC-MSC cell lines resting (left) or stimulated with IFN-γ and TNF-α (right). Expression levels range from red (low) to yellow (middle) to white (high). Evaluation of IDO1 (**B**) and IL4I1 (**C**) mRNA levels in four and five HNSCC-MSC primary cell lines, respectively. Cells were evaluated either resting or following overnight IFN-γ and TNF-a stimulation, each cytokine tested alone or in combination. Results are expressed as mean + SD. (**D**) CD4+ T cell proliferation evaluated by 3H-TdR uptake. T cells were stimulated with agonist anti-CD3+anti-CD28 antibodies in absence or presence of different concentrations of HNSCC-MSC. Co-cultures were performed in medium alone (white columns), in presence of catalase (1000 U/mL, gray columns) or 1-methyl-tryptophan (500 μM, black columns). Results are expressed as mean of cpm + SD from seven independent experiments. * *p* < 0.05; ** *p* < 0.01 with Student’s *t* test.

From the analysis of our already published HNSCC-MSC transcriptome data [8,9] we observed that among the genes mostly upregulated by IFN-γ and TNF-α stimulation there were IDO1 and IL4I1 (Figure 1A). Upregulation of IDO1 was expected, given that its expression has already been described in HNSCC-MSC and its known dependency on IFN-γ signaling [7]. In order to confirm transcriptome data, we performed a quantitative Real-Time PCR evaluation of IDO1 and IL4I1 mRNA expression levels in HNSCC-MSC cells treated or not with IFN-γ and TNF-α, each cytokine tested alone or in combination. As shown in Figure 1B, IFN-γ significantly upregulated IDO1 expression, while TNF-α alone had no effect. Of note, when TNF-α was added in combination to IFN-γ, it exerted a synergistic effect, allowing a further increase in IDO1 mRNA expression. Regarding IL4I1, IFN-γ alone had no statistically significant effect, while TNF-α significantly upregulated its expression (Figure 1C). As for IDO1, the combined activity of IFN-γ and TNF-α maximized IL4I1 expression. Finally, to functionally test the relevance of IL4I1 expression on the immunosuppressive activity of HNSCC-MSC, we set up a proliferation assay. CD4+ T cells, obtained from PB of healthy donors, were stimulated with agonist anti-CD3 and anti-CD28 antibodies and their proliferation was assessed by 3H-TdR uptake (Figure 1D). The proliferation rate was significantly reduced by the addition in culture of HNSCC-MSC in a dose-dependent manner, confirming their potent immunosuppressive effect. As expected, inhibition of IDO1 via 1-methyl-tryptophan addition completely restored T cell proliferation, and this phenomenon occurred even at high MSC concentration (1:5 ratio) (Figure 1D). Inhibition of IL4I1 activity by neutralizing H_2_O_2_ via catalase had also a positive effect on the restoration of T cell proliferation, although not as much as 1-methyl-tryptophan. Indeed, we observed a significant increase in T cell proliferation only at 1:10 ratio (Figure 1D). However, it should be noted that 1-methyl-tryptophan inhibits also the IL4I1-mediated catabolism of tryptophan, thus acting at the same time on both enzymes [10].

Here, we report that MSC are a source of IL4I1 in HNSCC microenvironment. Differently from IDO1, IL4I1 is secreted, thus it can exert its activity in a paracrine fashion. Indeed, IL4I1-derived catabolites H_2_O_2_ and I3P can affect immune cells and tumor cells, respectively. As already stated, H_2_O_2_ suppresses TCR expression, thus leading to impaired T cell activation and expansion. I3P instead increases tumor cell malignancy in an AhR-dependent mechanism, promoting their motility and metastases [4]. Following promising data obtained in preclinical models, IDO1 inhibitors are currently under consideration for clinical use in cancer patients, especially in combination with other immune-checkpoint blockers [11]. However, clinical trials failed to support a role for IDO1 targeting in cancer, so far [11]. A possible explanation comes from the evidence that other enzymes are involved in amino-acid catabolism in the tumor microenvironment. Indeed, targeting IDO1 alone may not be sufficient to completely block the amino-acid catabolism by cancer- and stromal-cells. The finding that IL4I1 can also act on these pathways suggests that additional inhibitors targeting this enzyme may be clinically relevant. The finding that IDO1 and IL4I1 are both expressed in cancer cells [4] and in stromal cells (MSC), further supports this observation. Moreover, IDO1 expression has been proposed as a candidate prognostic factor for clinical outcome in HNSCC patients [12]. IL4I1 may have a similar prognostic impact, thus future studies investigating this aspect should be considered. A major limitation of this study is that only in vitro data have been produced, on a limited number of samples. Thus, additional studies in in vivo models are needed to confirm these observations. If confirmed, these data would open new ways to therapeutic strategies aimed to contemporary block several enzymes involved in amino-acid catabolism. This approach may be helpful to contemporary interfere with tumor cell suppressive properties and restore an efficacious anti-tumor immunity.

## 4. Conclusions

In conclusion, we demonstrated that, in response to IFN-γ and TNF-α, HNSCC-MSC express the immunosuppressive enzyme IL4I1, which can suppress T cell proliferation.

## Data Availability

The data presented in this study are openly available at [9].
